# The Influence of Micronization on the Properties of Black Cumin Pressing Waste Material

**DOI:** 10.3390/ma17112501

**Published:** 2024-05-22

**Authors:** Renata Różyło, Grzegorz Gładyszewski, Dariusz Chocyk, Dariusz Dziki, Michał Świeca, Arkadiusz Matwijczuk, Klaudia Rząd, Dariusz Karcz, Sławomir Gawłowski, Monika Wójcik, Urszula Gawlik

**Affiliations:** 1 Department of Food Engineering and Machines, University of Life Sciences in Lublin, 28 Głęboka Str., 20-612 Lublin, Poland; slawomir.gawlowski@up.lublin.pl (S.G.); monika.wojcik@up.lublin.pl (M.W.); 2 Department of Applied Physics, Lublin University of Technology, 20-618 Lublin, Poland; g.gladyszewski@pollub.pl (G.G.); d.chocyk@pollub.pl (D.C.); 3 Department of Thermal Technology and Food Process Engineering, University of Life Sciences in Lublin, 31 Głęboka St., 20-612 Lublin, Poland; dariusz.dziki@up.lublin.pl; 4 Department of Biochemistry and Food Chemistry, University of Life Sciences in Lublin, 8 Skromna St., 20-704 Lublin, Poland; michal.swieca@up.lublin.pl (M.Ś.); urszula.gawlik@up.lublin.pl (U.G.); 5 Department of Biophysics, University of Life Sciences, 20-950 Lublin, Poland; arkadiusz.matwijczuk@up.lublin.pl (A.M.); klaudia.rzad@up.lublin.pl (K.R.); 6 Department of Cell Biology, Maria Curie-Sklodowska University, Akademicka 19, 20-033 Lublin, Poland; dariusz.karcz@pk.edu.pl; 7 Department of Chemical Technology and Environmental Analytics, Cracow University of Technology, 31-155 Krakow, Poland

**Keywords:** black cumin, pressing waste, micronization, particle size, FTIR spectroscopy, X-ray diffraction, antiradical activity

## Abstract

The purpose of this study was to investigate the effect of micronization on the characteristics of black cumin pressing waste material. The basic composition, amino acid, and fatty acid content of the raw material—specifically, black cumin pressing waste material—were determined. The samples were micronized in a planetary ball mill for periods ranging from 0 to 20 min. The particle sizes of micronized samples of black cumin pressing waste material were then examined using a laser analyzer, the Mastersizer 3000. The structures of the produced micronized powders was examined by X-ray diffraction. Additionally, the FTIR (Fourier-transform infrared) spectra of the micronized samples were recorded. The measurement of phenolic and antiradical properties was conducted both before and after in vitro digestion, and the evaluation of protein digestibility and trypsin inhibition was also conducted. The test results, including material properties, suggest that micronization for 10 min dramatically reduced particle diameters (d50) from 374.7 to 88.7 µm, whereas after 20 min, d50 decreased to only 64.5 µm. The results obtained using FTIR spectroscopy revealed alterations, especially in terms of intensity and, to a lesser extent, the shapes of the bands, indicating a significant impact on the molecular properties of the tested samples. X-ray diffraction profiles revealed that the internal structures of all powders are amorphous, and micronization methods have no effect on the internal structures of powders derived from black cumin pressing waste. Biochemical analyses revealed the viability of utilizing micronized powders from black cumin pressing waste materials as beneficial food additives, since micronization increased total phenolic extraction and antiradical activity.

## 1. Introduction

Various stages of food production and processing in the agri-food sector worldwide generate massive amounts of by-products and waste. Typically, this biomass is discarded, which in turn may cause serious environmental issues [1]. On the other hand, food waste can be reused to generate economic benefits, and one of the most intriguing strategies in this field is the processing of food by-products into edible materials [2]. Properly processed food by-products can be reused as food ingredients, dietary supplements, or pharmaceuticals [3]. For instance, a relatively high flavonoid or phenol content is observed in fruit or vegetable waste, while various bioactive peptides are present in the main wastes of the dairy industry [4]. As a result, the chemical compositions and contents of biologically active compounds in such by-products must be known [3]. 

It has been established that black cumin (*Nigella sativa*) seeds contain biologically active constituents [5]. Numerous studies have reported on the pro-health properties of these seeds and their potential usage in diets to prevent diabetes, dyslipidemia, hypertension, respiratory diseases, inflammatory diseases, and cancer [6]. Furthermore, *Nigella sativa* seeds are known to possess immune-stimulating, stomach-protective, liver-protective, kidney-protective, and neuroprotective properties. Therefore, black cumin, and particularly its seed oil, are used in traditional medicine [6]. 

Pressing waste, or cake, is another by-product of seed processing. For instance, the pressing waste from black cumin seed oil production can also be used as a food additive [7]. Recent research indicates that black cumin cakes have a high nutritional value because they contain proteins, phenolics, essential amino acids, and bioactive compounds. Meanwhile, significant quantities of these by-products are not utilized effectively, which may potentially result in economic loss and environmental pollution [8,9]. Due to its beneficial properties, cumin cake is used as a valuable addition to various functional bakery products [7] or fermented non-dairy beverages [10]. This product’s functional characteristics can be enhanced through various techniques, including micronization.

Micronization is a novel technical method used in food manufacturing to reduce particle sizes to micro- and even nanometers. There are several micronization methods, including fine grinding in a ball mill. The efficacy of a ball mill in lowering the particle sizes of various plant materials has been demonstrated [11,12]. A previous study on grape pomace powders demonstrated that grinding these materials boosted the powders’ solubility while decreasing their ability to retain water and bind oil and cations. Furthermore, micronization improved the extraction of phenolic components and antioxidant capacity. The acquired results indicate that micronization alters the content of dietary fiber, boosting its potential application as a functional ingredient in the food sector [13]. Other studies have also shown that micronization improves the extraction of active substances [14,15]. These modifications differ between various raw materials, which necessitates the execution of an investigation in this direction.

To fulfil this need, the aim of the current work is to examine the influence of micronization on the properties of black cumin pressing waste material. In more detail, its nutritional and antioxidant properties, as well as changes occurring in the product at the molecular level, were investigated using X-ray diffraction and FTIR (Fourier-transform infrared spectroscopy). 

## 2. Materials and Methods

### 2.1. Materials

The waste from black cumin seed pressing oil was obtained from the producer (Pliczko Farm, Woźniki, Poland). According to the manufacturer, this product was obtained by cold pressing at a temperature of about 38 degrees on a screw press. 

### 2.2. Chemical Analysis of Raw Materials

Black cumin pressing waste material (BCW) was analyzed for basic chemical composition, amino acid content, and fatty acid concentration. The protein content was determined using the Kjeldahl method (protein conversion factor Nx6.25) [16]; fat using the Soxhlet method [17]; ash using incineration [18]; moisture content using the drying method [19]; and dietary fiber using the Asp et al. method [20]. Subtracting the protein, fat, moisture, and dietary fiber content yielded the carbohydrate content. The calorific value of 100 g of sample was determined using the Atwater coefficients [21]. Fatty acid composition was determined using gas chromatography [22]. A chromatography system (Bruker 436GC, Vienna, Austria) equipped with a flame ionization detector (FID) was used to determine the fatty acid composition. A BPX 70 capillary column with nitrogen as the carrier gas was used to separate the fatty acid methyl esters. Most amino acids, except tryptophan, were measured following acid hydrolysis of proteins (6N HCl, 110 °C, 20 h) in a thimble hydrolyzer (Ingos, Prague, Czech Republic). To assess tryptophan content, the sample was treated through alkaline hydrolysis (Ba(OH)2, 110 °C, 20 h). The sample was then acidified with 6 N HCl, and a Na_2_SO_4_ solution was added. Cysteine was converted to cysteic acid and methionine to methionine sulfone via performic acid. After being prepared, the samples were subsequently loaded into the column of the AAA 400 amino acid analyzer (Ingos, Prague, Czech Republic). The identification of amino acids by ion exchange chromatography was conducted using a photometric detector (ninhydrin) at 570 nm for all amino acids and 440 nm for proline [23]. All measurements were repeated three times.

### 2.3. Micronization of Black Cumin Pressing Waste Material 

Black cumin pressing waste was ground for 60 s in a knife grinder before being micronized. Micronization, i.e., particle reduction, took place in a ball mill (600 rpm, Pulverisette 6, Fritsh, Germany) as previously described for 10 and 20 min [12]. During the preliminary tests, the micronization time was determined so that the particle sizes of the samples differed significantly. The minimum micronization time was chosen so that 50% of the particles (d50) had dimensions of less than 100 µm, and the subsequent multiple of this micronization time reduced particle size by at least 20%. As a result, three types of black cumin pressing waste material were obtained: one control sample (BCW), one micronized for 10 min (10MBCW), and one micronized for 20 min (20MBCW) [24]. 

### 2.4. Analysis of Particle Size

Particle size analyses of the control and micronized materials were performed using a laser analyzer, the Mastersizer 3000 (Malvern Instruments Ltd., Malvern, UK), with a dry dispersion attachment (Aero S), using the previously described methodology [25]. The mean particle sizes of volume D [4;3] [μm]) and the mean particle sizes of surface D [3;2] [μm] were obtained during the measurements. Particle sizes were also determined for 10% (d10), 50% (d50), and 90% (d90) of the total sample volume. 

### 2.5. X-ray Diffraction Analysis

The structural characteristics of the powders were analyzed through X-ray diffraction by an Empyrean X-ray diffractometer (PANalytical BV, Almelo, The Netherlands) with CuKα radiation (λ = 1.54056 Å), operated at a voltage of 40 kV with a current setting of 30 mA. The detection of diffracted X-rays was achieved using a proportional detector. Both the source divergence and the detector slit were set to a half-degree, and Soller slits were used to minimize spectral overlap. Measurements were conducted using a θ–2θ configuration at room temperature. The diffraction profiles were recorded with a step size of 0.01° and a counting time of 5 s per step to ensure adequate intensity measurement at each data point.

### 2.6. Measurements of Infrared Spectra—FTIR

The FTIR spectra of the selected black cumin pressing waste samples were recorded using an IRSprit spectrometer equipped with a ZnSe-based ATR (attenuated total reflection) extension (Shimadzu, Kyoto, Japan). The ZnSe crystal had the geometry (45°) required to provide optimum measurement conditions by facilitating multiple internal reflections of the laser beam during the procedure, and 24 scans were performed for each individual sample. The obtained spectral data were automatically averaged by the software. The extension crystal was kept clean throughout with the use of ultrapure solvents purchased from Sigma-Aldrich (Poznań, Poland). The spectra were measured within the range of 450–3600 cm^−1^, at a resolution of 2 cm^−1^. The obtained spectra were analyzed and processed using Grams AI software (Version 9.3) from ThermoGalactic Industries (Waltham, MA, USA). 

### 2.7. Extraction System for Biochemical Evaluations

For ethanol extraction, 0.5 g of raw material was homogenized with 5 mL of ethanol-water solution (1:1, *v*/*v*). The samples were shaken for 30 min at room temperature and then centrifuged for 15 min at 2558× *g*. The extraction procedure was repeated twice. The final samples were brought to 15 mL. 

### 2.8. Digestion In Vitro

In vitro digestion was carried out using a normalized INFOGEST procedure (www.cost-infogest.eu; accessed on 5 January 2024) [26]. To simulate mastication and gastrointestinal digestion, 2 g of sample was mixed with 2 mL of simulated salivary fluid [15.1 mmol/L KCl, 3.7 mmol/L KH_2_PO_4_, 13.6 mmol/L NaHCO_3_, 0.15 mmol/L MgCl_2_ (H_2_O)_6_, 0.06 mmol/L (NH_4_)_2_CO_3_, 1.5 mmol/L CaCl_2_, and α-amylase (75 U/mL)] and shaken for 10 min at 37 °C. The samples were then adjusted to pH 3 with 6 M HCl, suspended in 4 mL of simulated gastric fluid [6.9 mmol/L KCl, 0.9 mmol/L KH_2_PO_4_, 25 mmol/L NaHCO_3_, 47.2 mmol/L NaCl, 0.1 mmol/L MgCl_2_ (H_2_O)6, 0.5 mol/L (NH_4_)_2_CO_3_, 0.15 mmol/L CaCl_2_, and pepsin (2000 U/mL)] and shaken for 120 min at 37 °C. After simulated gastric digestion, the samples were adjusted to pH 7 with 1 M NaOH and suspended in 8 mL of simulated intestinal fluid [6.8 mmol/L KCl, 0.8 mmol/L KH_2_PO_4_, 85 mmol/L NaHCO_3_, 38.4 mmol/L NaCl, 0.33 mmol/L MgCl2 (H_2_O)_6_, 0.15 mmol/L CaCl_2_, 10 mmol/L bile extract, and pancreatin (2000 U/mL)]. The generated samples were subjected to in vitro intestinal digestion for 120 min. After digestion, the samples were centrifuged for 15 min at 6900× *g*. The supernatants were then combined with an equivalent volume of methanol. 

### 2.9. Phenolics and Antiradical Properties

#### 2.9.1. Total Phenolic Content

The Folin–Ciocâlteu reagent [27] was used to quantify total phenolics, which were expressed as gallic acid equivalents (GAE) in mg per gram of dry sample. Briefly, 10 μL of the sample was mixed with 10 μL of distilled water and 40 μL of the Folin–Ciocâlteu reagent (diluted with water 1:5). After 3 min, 250 μL of 10% Na_2_CO_3_ was added to the mixture. The contents were combined and left to stand for 30 min. The absorbance at 725 nm was measured using a Biotek Synergy H1 Hybrid Microplate Reader (ThermoFisher Sciences, Waltham, MA, USA) in comparison to a blank sample (ethanol–water solution (1:1, *v*/*v*) was used instead of plant extract).

#### 2.9.2. Ability to Quench ABTS Radicals

The tests were conducted using the ABTS decolorization assay [28]. The ABTS radical cation (ABTS+•) was formed by reacting 7 mmol L^−1^ stock solution of ABTS with 2.45 mmol L^−1^ potassium persulphate (final concentration). The ABTS+• solution was diluted with distilled water, resulting in an absorbance of 0.38 ± 0.05 at 734 nm. After adding 10 µL of sample to 250 µL of ABTS+• solution, absorbance was measured using a Biotek Synergy H1 Hybrid Microplate Reader (ThermoFisher Sciences, Waltham, MA, USA) against a blank sample after 1 h. The free radical scavenging ability was expressed as IC_50_ in mg/mL.

#### 2.9.3. DPPH Measurements

The DPPH radical scavenging activity was measured according to the approach reported by Brand-Williams et al. [29]. The samples were exposed to the stable DPPH radical in an ethanol solution. Ten µL of each sample were added to 250 µL of DPPH solution, and absorbance was measured using a Biotek Synergy H1 Hybrid Microplate Reader (ThermoFisher Sciences, Waltham, MA, USA) against the blank sample after 1 h. The scavenging ability of free DPPH radicals was expressed as IC50 in mg/mL. 

### 2.10. Protein Digestibility

The determination of the in vitro digestibility of proteins was performed according to Świeca and Baraniak [30]. Total proteins were extracted using 1 M NaOH (250 mg/8 mL) and measured according to the methods described by Bradford [31]. Undigested proteins were counted as a sum of protein in pellets and fluids obtained after digestion in vitro. The pellets were washed with 80% ethanol to remove free amino acids and peptides and extracted with 1 M NaOH. Protein contents in the fractions and fluids obtained after digestion (after appropriate corrections for the components of digestive fluids were made) were measured according to Bradford and expressed in bovine serum albumin (BSA) equivalents. The protein digestibility was calculated by subtracting the ratio of indigestible protein to total protein, and the result was multiplied by 100. 

### 2.11. Inhibition of Trypsin Activity

Trypsin activity was measured using Nα-Benzoyl-L-arginine 4-nitroanilide hydrochloride as a substrate [32]. The reaction mixture contained 220 µL of 100 mmol/L TRIS-HCL buffer pH 7.6, 10 µL of the enzyme (10 µg/mL, α-trypsin from bovine pancreas 1888 BAEE U/mg, T7409 Sigma-Aldrich, Poland), and 20 µL of the substrate (20 mmol/L Nα-Benzoyl-L-arginine 4-nitroanilide hydrochloride in DMSO, B3133, Sigma-Aldrich). The change in absorbance (after 3 min) was measured at 410 nm using a BioTek Epoch microplate reader. For the inhibition studies, the enzyme was incubated for 10 min with 10 µL of the studied extract (50 mg extracted with 1 mL of PBS) before adding the substrate. One unit released 1 μmole of 4-nitroanilide per minute at pH 7.6 at 37 °C. One inhibitory unit (IU) is defined as an inhibitor of activity that stops 1U of enzyme activity. The activity was expressed in IU per gram of powder.

### 2.12. Statistical Analysis

Means and standard deviations were calculated as a result of all analyses carried out in triplicate. ANOVA and Tukey’s test were used to determine the significance of differences (α = 0.05) between the means (Statistica 12.0, StatSoft, Krakow, Poland). Different outcomes are marked with different letters (a, b, c, etc.). 

## 3. Results

### 3.1. Chemical Characterization of Raw Material

The raw material for this research was black cumin pomace (BCW) obtained from the industry by cold pressing. The basic chemical composition of this pomace is given in Table 1. In previous studies, the protein content of black cumin seeds was about 20%, and the oil content was about 35% [7]. Since seed pomace is obtained after pressing the oil, the protein content increases and the oil content decreases. Data from the literature indicate fluctuations in the contents of protein, fat, and fiber depending on the variety and growing conditions of black cumin [5].

The amino acids content data (Table 2) show that the black cumin pomace mostly contained glutamic acid (Glu), followed by asparagine (Asp), arginine (Arg), proline (Pro), glycine (Gly), leucine (Leu), threonine (Thr), valine (Val), serine (Ser), alanine (Ala), lysine (Lys), phenylalanine (Phe), tyrosine (Tyr), isoleucine (Ile), methionine sulfone (Sulf met), cysteic acid (Cys ac), histidine (His), and tryptophan. Leucine was the most abundant essential amino acid, followed by threonine, valine, lysine, phenylalanine, isoleucine, and methionine sulfone. The amounts of histidine and tryptophan were the lowest among all the amino acids. The total amount of essential amino acids was 91 mg∙g^−1^. Arginine was the most prevalent of the conditionally essential amino acids, followed by proline, glycine, tyrosine, and cysteic acid. If the non-essential amino acids are considered, the most prevalent was glutamic acid, followed by asparagine, serine, and alanine. Previous research [23] on *Salvia hispanica* and *Ocimum tenuiflorum* seeds showed that most of the amino acids were below 10 mg/g. Glutamic acid was also the most abundant in these seeds, but the amount was much lower, in the range of 31.6 to 36.0 mg/g. The arginine content of *Salvia Hispanica* and *Ocimum tenuiflorum* seeds was close to the value found in black cumin pomace, i.e., 20.4 and 20.5 mg/g, respectively.

Among the fatty acids identified (Table 3) in BCW, the highest amount recorded was the sum of linoleic acid and linolelaidic acid. The sum of oleic acid and elaidic acid was lower—at less than half of that of linoleic and linolelaidic acid—followed by palmitic acid. The remaining fatty acids were in amounts below 1 g/100 g. Previous laboratory experiments on black cumin pomace revealed that linoleic acid was present in the highest concentration, followed by oleic acid and palmitic acid. The findings of other authors also revealed a high proportion of linoleic, oleic, and palmitic acids. Petroselinic acid was also found in high concentrations [33].

### 3.2. Particle Size Results of Black Cumin Micronized Powders

Table 4 presents the results of the powder particle sizes after micronization. Compared to the control sample (BCW), a significant reduction in particle size was observed after the micronization process. A total of 10 min of micronization resulted in a more than 4-fold decrease in the d50 particle size from 374.7 to 88.7 µm. Extending the micronization period from 10 to 20 min significantly decreased the d50 value, although only by about 27% to 64.5 µm. The average dimensions of the particles on the surface D [3;2] and volume D [4;3] decreased by more than three times, from 195.7 µm to 64.2 µm, and from 446 to 137 µm, respectively; in addition, D [3;2] decreased to the level of 44.1 µm, and D [4;3] to 88 µm. In previous experiments on raspberry pomace micronization [12], 10 min was sufficient to achieve a considerable particle reduction. The particle sizes obtained from micronized raspberry pomace powders were significantly smaller than in previous research, which was undoubtedly influenced by the kind and the mechanical qualities of the treated tissue. Other studies [34,35] also show that micronization causes different degrees of fragmentation, which may depend on the type of material being processed.

### 3.3. FTIR Spectroscopic Analysis of Black Cumin Micronized Powders

**Figure 1:**
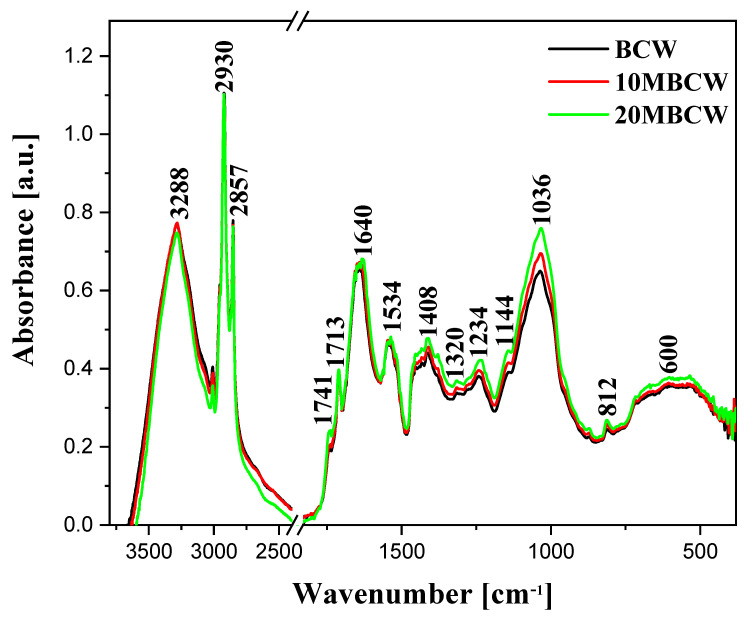
FTIR spectra of the black cumin samples subjected to micronization.

The effects of black cumin pomace micronization were examined by FTIR spectroscopy (Figure 1) and are presented in Table 5, together with the assignment of bands originating from the specific functional groups [36,37,38,39,40,41,42,43,44]. 

The cellulose and hemicellulose molecules, and particularly their valence hydroxyl groups, gave rise to broad bands with maxima at ~3290 cm^−1^ [42,44]. These groups are involved in the formation of hydrogen bonds between the smaller cellulose molecules, which were the main component of fiber present in the samples tested. Another set of bands present in this region were characteristic of asymmetric and symmetric stretching vibrations in CH_2_ groups at ~2930 and 2857 cm^−1^, respectively. In samples subjected only to micronization, the intensities of those bands slightly increased. The most prominent bands observed in the fingerprint region were observed at ~1740 and 1713 cm^−1^, assigned to C=O stretching vibrations characteristic of carbonyl groups present in fat and protein residue molecules at ~1740 and 1713 cm^−1^ [44]. Also, the highly intensive, and thus well-resolved, band with the maximum at ~1640 cm^−1^ was observed in this region, most likely due to the bending vibrations of the hydroxyl moieties [36,44]. This band corresponds to vibrations of the Amide I grouping in protein structures, significant amounts of which were present in the samples analyzed. The intensity of this band clearly increased upon micronization. 

Significant intensity changes were observed in bands positioned below 1600 cm^−1^ such as the intensity of band at 1534 cm^−1^, characteristic of ν(C=C) vibrations [36,37,38,39,40,41,42,43]. The discussed region corresponds well with the characteristics of protein systems associated with the vibrations of the Amide II structure. The band with the maximum at ~1408 cm^−1^ corresponds to the deformation vibrations in -CH_2_- groups, while the band at 1320 cm^−1^ is characteristic of δ(C-H) vibrations, which may be enhanced by δ(O-H) vibrations. These groups are present in cellulose and hemicellulose molecules, which constitute the main building materials of the analyzed samples. An increase in the intensities of the bands at 1234 and ~1144 cm^−1^ was also observed. These are characteristic of the respective δ(-OH in plane), δ (CH_2_), and δ (C-H) groups, as well as the stretching vibrations of the C-O-C system in cellulose and hemicellulose molecules present in fiber. Moreover, the band with the maximum at 1234 cm^−1^ corresponds to the vibrations of the Amide III structure, which is another element characteristic of the protein structures. Furthermore, notable changes were observed in the area near 1036 cm^−1^. These signals are characteristic of the stretching C-O vibrations, originating particularly from the C-O-C system in cellulose and hemicellulose. These bands demonstrated a significant increase in intensity in samples subjected to micronization.

The region below 1000 cm^−1^ was occupied by bands associated with vibrations of the crystalline material studied. For instance, conformation changes associated mainly with the vibrations of β-1,4-glycoside bonds in cellulose molecules were present in this region [44]. Furthermore, a sharp and low-intensity band with the maximum at approx. 812 cm^−1^ was observed, as well as a wide band at ~600 cm^−1^. Compared to those of the reference sample, their intensities were considerably higher, though no significant differences were observed between samples subjected only to micronization. Nonetheless, the variations in terms of vibration intensity observed in this region indicate changes taking place in bonds between individual materials within the molecules of fiber structures [34,35,45]. 

In conclusion, the FTIR results obtained, particularly the changes in the intensities and shapes of individual bands, suggest a strong impact caused by the molecular characteristics of the analyzed material. It is worth emphasizing that the intensity changes in particular were clearly visible in samples subjected only to time-dependent micronization. The micronization of black cumin pressing waste resulted in the cleavage of intramolecular hydrogen bonds between cellulose and hemicellulose, which are present in large quantities in the samples tested. This, in turn, caused amorphic growth of cellulose structures [34,35,45]. This process was reflected by changes in the intensities of bands characteristic of stretching vibrations in hydroxyl groups, as well as a notable increase in the intensity of the band with the maximum at 1640 cm^−1^. It was also reflected by the intensity changes of bands at 1408, 1234, and 1036 cm^−1^. As reported in the literature, micronization primarily tends to cleave the amorphic regions on ordered surfaces of crystalline substances [34,35,45]. Hence, the relatively stiff and ordered structure of cellulose is likely to be damaged during a long-lasting grinding process. It is noteworthy that a significant increase in band intensity was also observed for vibrations characteristic of proteins, namely Amide I, II, and III structures—i.e., bands with the maxima at, respectively, 1640, 1534, and 1234 cm^−1^.

### 3.4. X-ray Diffraction Profiles of Black Cumin Micronized Powders

**Figure 2:**
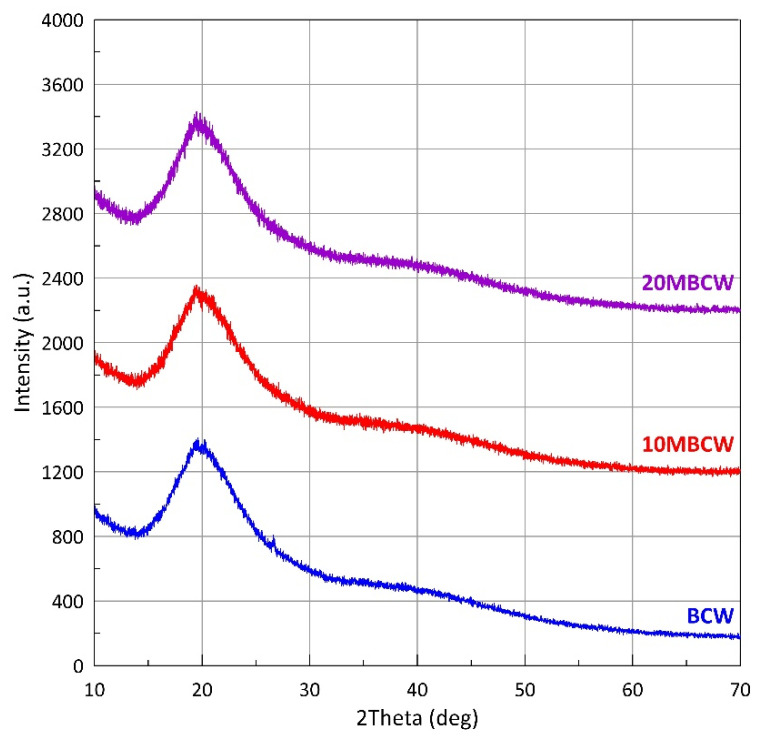
X-ray diffraction profiles of control and micronized powders obtained from black cumin pressing waste.

Figure 2 shows the diffraction profiles obtained for the control sample (BCW) and the samples micronized for 10 and 20 min (10MBCW and 20MBCW, respectively). From the results for all samples shown in the figure, it is clear that the internal structures of all powders are amorphous, as indicated by the main broad peak present in each diffraction profile at around 20°. The obtained profiles were fitted to obtain the position of the main peak and the corresponding average interatomic distance for each sample (Table 6). The obtained values of the mean interatomic distances do not show any change tendency, and the difference between the obtained values is small (maximum difference is equal to 0.005 nm). The above demonstrates that micronization processes do not alter the internal structures of the powders derived from black cumin pressing waste.

### 3.5. Biochemical Properties of Black Cumin Micronized Powders

This study found that micronization of black cumin pomace improves the sample’s biochemical characteristics (Table 7). The total content of phenolic compounds (TPC) in the black cumin pomace preparations micronized for 10 and 20 min was more than twice as high (23.71; 24.68 mg GAE/g dry matter) as in the control sample (11.53 mg). After digesting the samples, a significant increase in TPC, more than two times greater, was also achieved. The EC50 index was used to measure the antiradical activity of the micronized product after 10 min. The antiradical activity of the preparation micronized for 10 min, as measured by the EC50 index against ABTS before and after digestion, was particularly low, at 0.91 and 1.15 mg d.m./mL, and much lower than that of the control sample (1.18; 1.77 mg d.m./mL). Micronizing the sample for 20 min had a negative impact on this indicator, thereby increasing its value. When the micronization duration is extended, the temperature inside the working chamber rises, potentially leading to the breakdown of active compounds. Several process parameters can influence the degradation of active substances [46].

The antiradical activity of the preparation that was micronized for 10 and 20 min, as measured by the EC50 index against DPPH, exhibited a favorable decline with increasing micronization time. The EC50 index indicated that the antiradical activity of the preparation decreased significantly with increasing micronization time, reaching a value of 1.92 mg d.m./mL after digestion—6.75 mg d.m./mL lower than the control sample, which had a very high value after digestion, up to 32.66 mg d.m./mL. Increasing the micronization time did not provide a substantial drop in this indicator. The 10 min method resulted in a considerable improvement in biochemical parameters over the control sample. Similar to this study, several investigations found that micronizing olive pomace enhances the bioaccessibility and antioxidant potential of phenolic compounds [15]. Furthermore, micronization of grape pomace enhances phenolic component extraction, particularly catechin and epicatechin, as well as antioxidant capacity, as measured by the ABTS and ORAC (Oxygen Radical Absorbance Capacity) assays [13]. The antioxidant potential of micronized raspberry pomace, as measured by ABTS and FRAP (Ferric Reducing Antioxidant Power), increased considerably after the micronization method was used [12]. Most available studies show an increase in antioxidant activity following the micronization process, which may be associated with an increase in the extractability of active chemicals. These modifications are different for various materials, and extending the micronization process does not result in dramatic changes; therefore, it is required to determine suitable micronization parameters for various materials.

**Figure 3:**
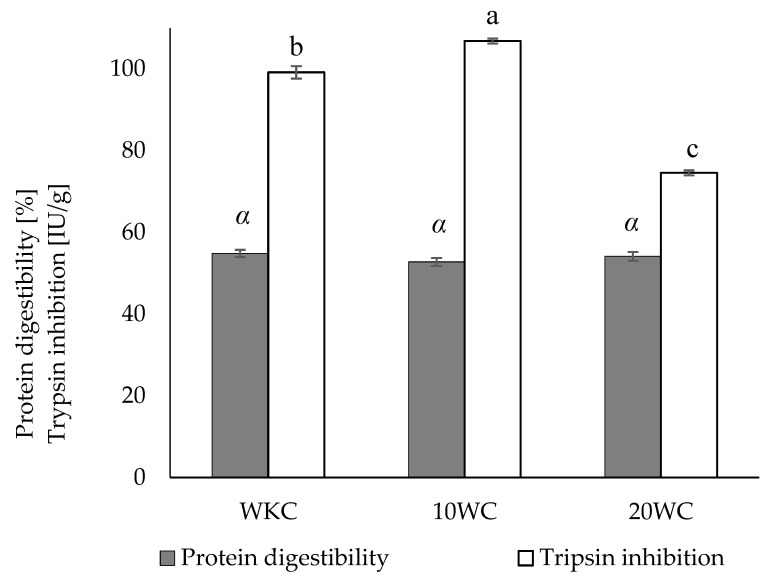
Protein digestibility and trypsin inhibition of micronized powders obtained from black cumin pressing waste. a, b, c values in the same series on the chart marked with different letters are significantly (α = 0.05) different.

When it comes to protein digestibility (Figure 3), there were no significant changes in this indicator following micronization for either 10 or 20 min compared to the control sample. Only after 20 min of micronization did trypsin inhibition significantly decrease; 10 min of micronization resulted in a minor but significant increase in trypsin inhibition. Other studies have found that the particle size of micronized soybean meal has no effect on protein or amino acid digestion [47]. Studies on ground maize show that particles larger than 595 µm have decreased crude protein digestibility in vitro. No such research has been carried out in smaller particle size ranges [48].

## 4. Conclusions

The micronization of black cumin pomace with a ball mill resulted in a considerable reduction in particle size. The control powder had a d50 size of 374.7 µm. The highest change, resulting in particles becoming nearly four times smaller, happened after 10 min of micronization (d50 = 88.7 µm).

The analysis of infrared spectra revealed the most significant changes in bands, with the maxima at ~3288, 1640, 1534, 1420–1200, and 1036 cm^−1^. The changes were related mainly to the intensities of the bands, but also to slight shifts thereof.

Micronization methods have little effect on the internal structures of powders derived from black cumin pressing waste.

This study discovered that micronization of black cumin pomace increases the samples’ biochemical properties (TPC, DPPH) both before and after in vitro digestion.

In conclusion, it should be noted that, after considering all parameters, the most optimal and sufficient time for the micronization of black cumin pomace is 10 min; a longer process does not produce any significant changes. Thus, micronizing black cumin pomace results in a product with improved antioxidant activity, which can be utilized as a beneficial food ingredient.

## Figures and Tables

**Table 1:** Basic chemical composition of black cumin pressing waste.

Parameter	Amount
Humidity (g/100 g)	5.87 ± 0.27
Protein (g/100 g)	29.86 ± 1.15
Fat (g/100 g)	23.05 ± 1.41
Ash (g/100 g)	5.25 ± 0.31
Fiber (g/100 g)	35.57 ± 1.13
Carbohydrates (g/100 g)	0.4
Calorific value (kcal/100 g)	400

**Table 2:** Amino acid composition of black cumin pressing waste.

	Kind of Amino Acid	Amount of Amino Acid (mg∙g^−1^)
Essential amino acids	Threonine (Thr)	12.9 ± 0.6
	Valine (Val)	12.3 ± 0.5
	Methionine sulfone (Sulf met)	8.9 ± 0.4
	Isoleucine (Ile)	9.5 ± 0.3
	Leucine (Leu)	16.1 ± 0.5
	Phenylalanine (Phe)	10.3 ± 0.6
	Histidine (His)	7.9 ± 0.3
	Lysine (Lys)	10.7 ± 0.4
	Tryptophan (Trp)	2.4 ± 0.1
Conditionally essential amino acids	Proline (Pro)	18.2 ± 0.9
	Glycine (Gly)	16.9 ± 0.9
	Cysteic acid (Cys ac)	8.7 ± 0.3
	Tyrosine (Tyr)	10.1 ± 0.4
	Arginine (Arg)	23.1 ± 1.0
Non-essential amino acids	Alanine (Ala)	11.4 ± 0.4
	Asparagine (Asp)	29.9 ± 1.4
	Serine (Ser)	11.8 ± 0.7
	Glutamic acid (Glu)	70.6 ± 2.3

**Table 3:** Composition of fatty acids in black cumin pressing waste (BCW).

Fatty Acids	Amount of Fatty Acid (g/100 g)
Essential fatty acids	
C 18:3n3 (α-linolenic acid)	0.046 ± 0.001
C 20:3n3 (cis-11, 14, 17-eicosatrienoic acid)	0.016 ± 0.001
C 18:2n6c+ C 18:2n6t (linoleic acid + linolelaidonic acid)	12.05 ± 0.531
Non-essential fatty acids	
C 6:0 (hexanoic acid)	0.007 ±0.000
C 8:0 (octanoic acid)	0.025 ± 0.001
C 10:0 (decanoic acid)	0.023 ± 0.001
C 12:0 (lauric acid)	0.009 ± 0.000
C 14:0 (myristic acid)	0.069 ± 0.002
C 15:0 (pentadecanoic acid)	0.009 ± 0.000
C 16:0 (palmitic acid)	2.81 ± 0.093
C 16:1n7 (palmitoleic acid)	0.048 ± 0.002
C 18:0 (stearic acid)	0.791 ± 0.021
C 18:1n9c+ C 18:1n9t (oleic acid + elaidic acid)	5.85 ± 0.303
C 20:0 arachidic acid	0.053 ± 0.002
C 20:1n15	0.065 ± 0.002
C 20:1n9 (cis-11-eicosenoic acid)	0.530 ± 0.034
C 22:0 (behenic acid)	0.053 ± 0.002
C 22:1n9 (erucic acid)	0.012 ± 0.000
C 23:0 (tricosanoic acid)	0.009 ± 0.000
SFA (Saturated fatty acid)	3.85
MUFA (mono unsaturated fatty acid)	6.50
PUFA (polyunsaturated fatty acid)	12.15
OMEGA 3 *	0.09
OMEGA 6 **	12.05
OMEGA 9 ***	6.39

* OMEGA 3 is the sum of the acids C18:3n3, C20:3n3, C 0:5n3, C22:6n3. ** OMEGA 6 is the sum of the acids C182n6c, C183n6, C202n6, C20:3n6, C20:4n6, C22:2n6. *** OMEGA 9 is the sum of the acids C18:1n9c, C20:1n9, C22:1n9, C24:1n9.

**Table 4:** Particle size characteristics of micronized powders obtained from black cumin pressing waste.

	D [3;2] (µm)	D [4;3] (µm)	d10 (µm)	d50 (µm)	d90 (µm)
BCW	195.7 ± 23.9 a	446 ± 37.4 a	83.8 ± 2.0 a	374.7 ± 6.9 a	808.7 ± 11.5 a
10MBCW	64.2 ± 1.5 b	137 ± 7.4 b	33.3 ± 1.0 b	88.7 ± 2.5 b	328.0 ± 32.1 b
20MBCW	44.1 ± 0.2 c	88.0 ± 2.5 c	22.6 ± 0.1 c	64.5 ± 0.4 c	162.1 ± 1.7 c

D [3;2]—average particle dimensions of the sample in the surface area; D [4;3]—average particle dimensions of the sample in the volume area; d10, d50, and d90—particle size below their value is 10, 50, and 90% of the total volume of the sample; BCW—control black cumin pressing waste; 10MBCW—black cumin pressing waste micronized for 10 min; 20MBCW—black cumin pressing waste micronized for 20 min. a–c values in the same column marked with different letters are significantly (α = 0.05) different.

**Table 5:** The assignment of FTIR absorption bands, registered within the spectral range of 450–3600 cm^−1^.

FTIR	Types and Origins of Vibrations
Positioning of Band [cm^−1^]	
3288	*(intra-)*molecular hydrogen bonding and ν(O-H) in H_2_O
2913	*asymmetric* and *symmetric*: ν(C-H) in CH_2_ and CH_3_
2845	
1736	ν(C=O) *free and hydrogen-bound*
1706	
1640	ν(C=C) and Amide I and δ(O-H) adsorbed H_2_O
1534	ν (C=C) and Amide II
1408	δ (CH_2_), δ (C-H) enhanced by δ (-OH in plane)
1312	δ(O-H) but mainly δ(C-H)
1234	δ(C-H) and asymmetric bridge oxygen stretching -OH *in-plane* bending and Amide III
1137	ν(*C-O-C*) and strong ν(*C-O*) and ring stretching modes and asymmetric in-phase ring stretching
1029	
806	*β-*linkage of cellulose/ring breathing and asymmetric *out of phase* stretching -OH *out-of-plane* bending and CH_2_ rocking
608	

ν—stretching vibrations, δ—deformation vibrations.

**Table 6:** The positions of the main peak and the corresponding average inter-atomic distance in each powder obtained from black cumin pressing waste.

	2θ (deg)	d-Space (nm)
BCW	19.90°	0.446
10MBCW	19.94°	0.446
20MBCW	19.86°	0.447

BCW—control black cumin pressing waste; 10MBCW—black cumin pressing waste micronized for 10 min; 20MBCW—black cumin pressing waste micronized for 20 min. The accuracy of determining the peak positions in the θ–2θ profiles was +/−0.05 degrees, which corresponds to an accuracy of +/−0.0011 nm in determining the average interatomic distances.

**Table 7:** Biochemical properties of micronized powders obtained from black cumin pressing waste.

Name of Sample	TPC [mg GAE/g d.m.]		ABTS [EC 50 mg/mL]		DPPH [EC 50 mg/mL]	
	Before Digestion	After Digestion	Before Digestion	After Digestion	Before Digestion	After Digestion
BCW	11.53 ± 0.17 aA	14.45 ± 0.21 aB	1.18 ± 0.01 aA	1.77 ± 0.04 aC	2.89 ± 0.14 aB	32.66 ± 2.42 aD
10MBCW	23.71 ± 1.08 bC	29.68 ± 1.33 bD	0.91 ± 0.00 bB	1.15 ± 0.01 cA	1.92 ± 0.01 bA	6.75 ± 0.20 bC
20MBCW	24.68 ± 1.01 bC	30.90 ± 1.46 bD	1.15 ± 0.03 aA	1.60 ± 0.05 bC	1.54 ± 0.06 cA	2.42 ± 0.02 cB

BCW—control black cumin pressing waste; 10MBCW—black cumin pressing waste micronized for 10 min; 20MBCW—black cumin pressing waste micronized for 20 min. a–c values in the same column marked with different letters are significantly (α = 0.05) different. A–D values in the same method (TPC, ABTS, DPPH) marked with different letters are significantly (α = 0.05) different.

## Data Availability

Data are contained within the article.
